# Purification of Spherical Graphite as Anode for Li-Ion Battery: A Comparative Study on the Purifying Approaches

**DOI:** 10.3390/mi15070827

**Published:** 2024-06-27

**Authors:** Tri Thien Vu, Duong Duc La, Long Vu Le, Trung Kien Pham, Minh Anh Nguyen, Tran Hung Nguyen, Trung Dung Dang, Myoung-Jin Um, Woojin Chung, Dinh Duc Nguyen

**Affiliations:** 1 Institute of Chemistry and Materials, Hanoi 10000, Vietnam; thienkqh140309@gmail.com (T.T.V.); duc.duong.la@gmail.com (D.D.L.); lelongvullv@gmail.com (L.V.L.); phamtrungkien88@gmail.com (T.K.P.); nguyentranhung28@gmail.com (T.H.N.); 2 School of Chemistry and Life Sciences, Hanoi University of Science and Technology, Hanoi 10000, Vietnam; anh.nm190665@sis.hust.edu.vn; 3 Department of Environmental Energy Engineering, Kyonggi University, Goyang 10285, Republic of Korea; mum@kyonggi.ac.kr; 4 Institute of Applied Technology and Sustainable Development, Nguyen Tat Thanh University, Ho Chi Minh City 700000, Vietnam

**Keywords:** batteries, Li-ion battery, spherical graphite, anode for batteries, graphite purification

## Abstract

Graphite is a versatile material used in various fields, particularly in the power source manufacturing industry. Nowadays, graphite holds a unique position in materials for anode electrodes in lithium-ion batteries. With a carbon content of over 99% being a requirement for graphite to serve as an electrode material, the graphite refinement process plays a pivotal role in the research and development of anode materials for lithium-ion batteries. This study used three different processes to purify spherical graphite through wet chemical methods. The spherical graphite after the purification processes was analysed for carbon content by using energy-dispersive X-ray (EDX) spectroscopy and was evaluated for structural and morphological characteristics through X-ray diffraction (XRD), scanning electron microscopy (SEM), and Brunauer–Emmett–Teller (BET) analyses. The analyses results indicate that the three-step process via H_2_SO_4_–NaOH–HCl cleaning can elevate the carbon content from 90% to above 99.9% while still maintaining the graphite structure and spherical morphology, thus enhancing the surface area of the material for anode application. Furthermore, the spherical graphite was studied for electrochemical properties when used as an anode for Li-ion batteries using cyclic voltammetry (CV) and galvanostatic charge–discharge (GCD) measurements. The results demonstrated that the purification process significantly improves the material’s capacity with a specific capacity of 350 mAh/g compared to the 280 mAh/g capacity of the anode made of spherical graphite without purification.

## 1. Introduction

Graphite is a versatile material widely applied across various industries today. It is a unique non-metal material possessing the advantages of a metal, such as good electrical and thermal conductivity and high mechanical strength [1]. Currently, graphite is extensively utilized in many fields, including but not limited to crucibles, foundry additives, mould coatings, lubricants, aerospace applications, and graphene [2,3,4,5]. One of graphite’s significant current and future applications is as an anode material for Li-ion batteries [6,7]. Graphite can be divided into two groups: natural graphite and synthetic graphite. Therefore, natural graphite is commonly used for anode electrodes due to the fact that it has lower processing costs than synthetic graphite while it retains advantages in terms of chemical and mechanical durability, electrical conductivity, and stability. Graphite has three forms: amorphous, flake, and vein [8]. Amorphous graphite has low electrical conductivity, making it the most common but lowest-quality form. Flake graphite has a large reserve, especially in Vietnam, where the primary deposits in Bao Ha, Lao Cai, are predominantly flake graphite [9]. Vein graphite has the highest quality and value compared to the other two types. However, vein graphite reserves are very scarce, making it difficult to supply the current demand for Li-ion batteries.

However, flake graphite has the disadvantage of having a small surface area, leading to a limited material capacity [10]. Therefore, to achieve the highest possible output capacity, the working surface area of the material needs to be improved, and forming a spherical shape from flake form is one of the potential directions. Flake graphite was first sphericalized in 2000 by Yoshio et al. [11]. The research team successfully rolled crushed flake graphite to create spherical materials with a diameter of about 20 µm, achieving an efficiency of around 30–50%. In addition to morphology, carbon content is also a significant factor influencing the suitability of materials for electrode applications in batteries [12]. To be used as an electrode material for Li-ion batteries, the carbon content typically needs to be above 99%, while natural flake graphite typically only has a carbon content ranging from 85 to 96%. Therefore, purifying graphite to a carbon content over 99% is considered a crucial process in applying spherical graphite for Li-ion batteries.

Many methods are used for purifying graphite, such as flotation, microwave, and gravity separation, etc. [13,14,15,16,17,18]. To achieve a carbon content above 99%, thermal and chemical methods are two commonly used methods nowadays [13]. However, the equipment used for thermal methods is complex and expensive, leading to relatively low economic efficiency. Therefore, in recent years, research has focused mainly on chemical methods for graphite purification. K. Zaghib et al. conducted a study comparing the efficiency of wet chemical methods and thermal methods [13]. Although the wet chemical methods resulted in a higher Si impurity content than the thermal methods, considering economic feasibility and scalability, the research findings also suggested that wet chemical methods are a promising direction for graphite purification processes.

The wet chemical refinement method involves using certain chemical types to dissolve the minerals’ impurities [19]. For graphite minerals, some commonly used chemicals for impurity removal are H_2_SO_4_, HNO_3_, and HCl acids [14,20,21]. Using these chemicals can yield graphite with a carbon content > 95%. Silicate impurities are the most difficult to handle in graphite, and HF is commonly used to remove this impurity group. However, HF is highly environmentally toxic when applied on an industrial scale [22]. Therefore, many alternative chemicals have been researched to remove silicate instead of HF, and NaOH is a potential cleaning agent. Some studies have utilized a mixed process of H_2_SO_4_/H_2_O_2_–NaOH–HCl to purify graphite, achieving purity levels of up to 99.68% [3]. Additionally, a two-step alkaline–acid cleaning process has been researched, with the carbon content reaching up to 98.6% [21]. However, most of the purifying processes focused on flake graphite.

This study explored chemical purifying methods for spherical graphite. The three selected processes designed for investigation were a three-step acid–alkali–acid process (H_2_SO_4_–NaOH–HCl), a two-step alkali–acid process (NaOH–H_2_SO_4_), and finally a two-step acid–acid process (H_2_SO_4_–HF). This research aimed to investigate and identify the optimal method for achieving a carbon content above 99% in spherical graphite while maintaining the spherical particle morphology for subsequent application as an anode electrode material for Li-ion batteries. The characterisations of spherical graphite before and after purification were carried out using SEM, EDX, XRD, and BET analyses. The electrochemical properties of anodes fabricated from purified spherical graphite were also studied and discussed.

## 2. Experimental Section

### 2.1. Materials

The materials used in this study were natural spherical graphite extracted and processed from the Bao Ha mine, Lao Cai Province, Vietnam. The chemicals used for the refining process included 98% H_2_SO_4_, 99% NaOH, 36.5% HCl, and 20% HF. Eight percent PdVF, LiPF_6_, and Super P were purchased from Xilong Chemicals (Shanghai, China). All chemicals were utilized as received without any further purification.

### 2.2. Spherical Graphite Purification

**Figure 1:**
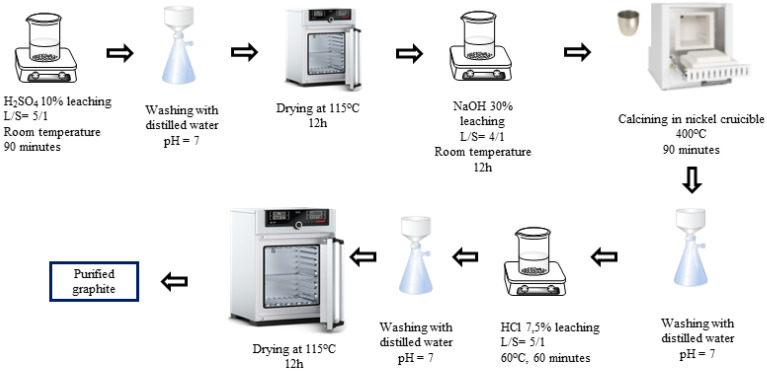
Schematic purifying process using H_2_SO_4_, NaOH, and HCl.

Three purifying processes were carried out in this study:

H_2_SO_4_-NaOH-HCl (P-I) purifying process (Figure 1): The spherical graphite was immersed in an H_2_SO_4_ 10% solution with a solid/liquid mass ratio of 1/5. The mixture was stirred by using a magnetic stir at room temperature for 90 min. Then, the slurry was washed and filtered with distilled water until it reached a neutral pH of 7, and the solid portion was collected. The solid was then dried at 115 °C for 12 h. After drying, the material was further immersed in a 30% NaOH solution with a solid/liquid mass ratio of 1/4. The graphite was evenly dispersed in the alkali by stirring with a magnetic stirrer for 12 h and was calcinated by using a Nabertherm P330 device at 400 °C for 90 min. The resulting mixture was washed, filtered, and then dispersed in an HCl 7.5% solution for 60 min. After rinsing with HCl, the graphite–HCl slurry was washed and filtered with distilled water until the pH reached 7, and the solid portion was collected. The solid product (P-I) was dried at 115 °C for 12 h.

**Figure 2:**
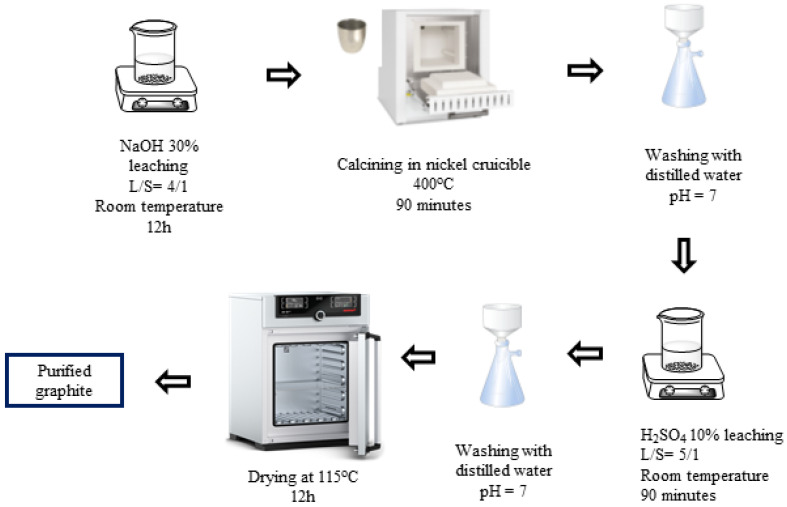
Schematic purifying process using H_2_SO_4_ and NaOH.

H_2_SO_4_–NaOH (P-II) purifying process (Figure 2): First, the spherical graphite was immersed and stirred in a NaOH 30% solution for 12 h with a solid/liquid mass ratio of 1/4, and then the mixture was calcined at 400 °C for 90 min. The product, after calcination, was washed, filtered, and then immersed in an H_2_SO_4_ 10% solution with a solid/liquid mass ratio of 1/5 at room temperature for 90 min. After the acid leaching step, the mixture was washed and filtered with distilled water until the pH reached 7, and the solid portion was collected. The solid material (P-II) was dried at 115 °C for 12 h.

**Figure 3:**
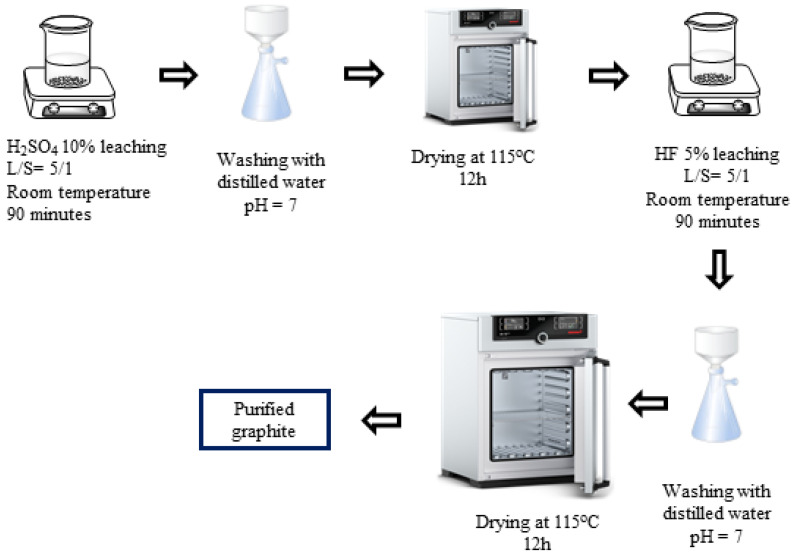
Schematic purifying process using H_2_SO_4_ and HF.

H_2_SO_4_–HF (P-III) purifying process (Figure 3): In the third process, spherical graphite was leached in an H_2_SO_4_ 10% solution with parameters similar to those in process 1. Then, the solid material, after drying, was dispersed in an HF 5% solution for 90 min with a solid/liquid mass ratio of 1/5. After the acid leaching step, the mixture was washed and filtered with distilled water until the pH reached 7, and the solid material was collected. The purified graphite (P-III) was finally dried at 115 °C for 12 h.

### 2.3. Characterizations

The spherical graphite before and after the purifying processes was analysed and evaluated for chemical composition, structural characteristics, and morphology by using energy-dispersive X-ray spectroscopy (EDX), X-ray diffraction (XRD), scanning electron microscopy (SEM), and Brunauer–Emmett–Teller (BET) analysis.

### 2.4. Electrochemical Investigation

0.5 g of the spherical graphite before (SG1) and after (SG2) purification was mixed with a binder PVDF and a conductive supporter Super P in a mass ratio of 8:1:1. After the mixing step, the mixture was rolled onto a copper foil. Then, the electrode sheet was dried at 50 °C for 12 h and vacuum-dried for 8 h at 90 °C. After that, the sheets were cut into the circle electrodes with a diameter of 1.1 cm. These electrodes were assembled into the coin cell battery model CR 2032, and the electrochemical characteristics were evaluated through cyclic voltammetry (CV) and galvanic charge discharge (GCD) measurements using a WonaTech WBCS 3000 device (WonaTech, Seoul, Republic of Korea).

## 3. Results and Discussion

### 3.1. Selection of Purification Process

**Figure 4:**
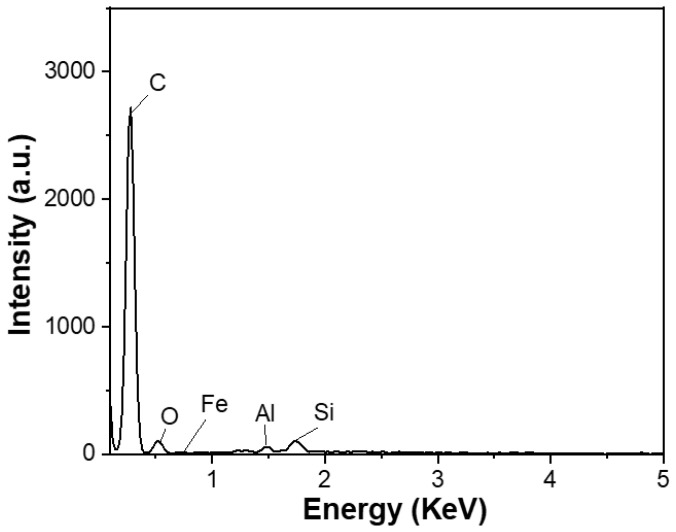
EDX spectrum of the spherical graphite before purification.

The carbon content of the spherical graphite before and after purification was evaluated using energy-dispersive X-ray spectroscopy (EDX). Before refinement, the material (SG1) had a carbon content of about 90% and was accompanied by impurities such as Fe (0.57%), Al (0.32%), Si (1.48%), and O (7.63%), as shown in Figure 4. Tran Thi Hien also analysed the graphite composition from the Bao Ha mine, Lao Cai, and published the impurity composition in the material, including oxides of Al, Fe, and Si [20]. Additionally, according to Wang et al. [21], who used the XRF method, graphite’s main impurities exist in oxides, predominantly Fe_2_O_3_, Al_2_O_3_, and SiO_2_.

**Figure 5:**
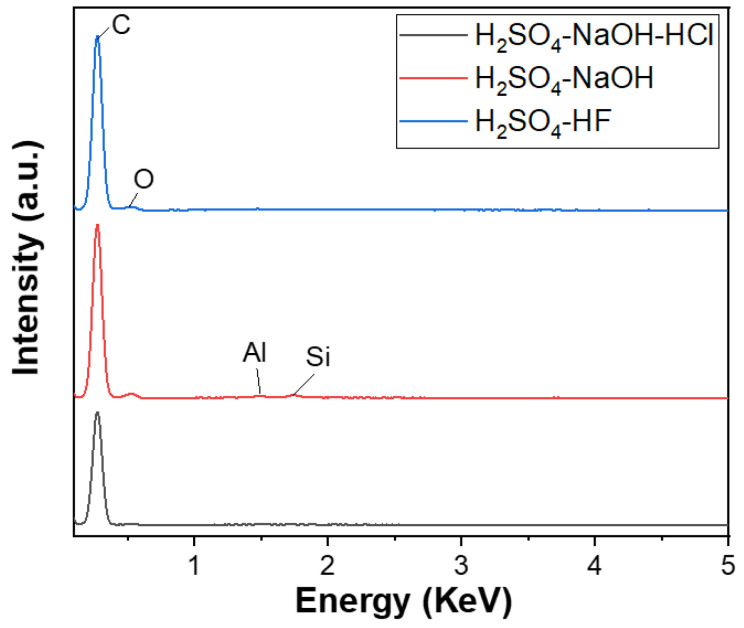
EDX spectra of spherical graphite after purification using various processes.

The EDX analysis results of three graphite samples after purifying P-I, P-II, and P-III, corresponding to three cleaning processes, are shown in Figure 5. The EDX spectrum of the first cleaning process’s product, corresponding to the three main cleaning steps using H_2_SO_4_–NaOH–HCl, achieved a carbon content of approximately 100%. The second process’s product with two main refining agents, NaOH–H_2_SO_4_, also reached a carbon content of 99.09%, where Fe (0.28%), Si (0.34%), Al (0.28%), and O impurities still existed. However, the content of these impurities were significantly decreased. The third process’s product, using H_2_SO_4_ with HF as the primary cleaning agent, also had a carbon content of 99.86%, with only a small amount of Al impurities, about 0.14%. Thus, a three-step purifying process using H_2_SO_4_–NaOH–HCl is considered the most suitable for obtaining high-quality spherical graphite for the Li-ion battery application.

H_2_SO_4_ and HCl were evaluated for their ability to effectively clean impurities such as Al and Fe [16,17,18,19,20,21,22]. Both acids exhibit strong oxidizing properties; however, HCl is more effective because when dissolving impurities, chloride salts tend to dissolve more readily than some sulfate salts [13]. In the first step of the acid immersion process, H_2_SO_4_ was used due to having a lower cost than HCl while maintaining similar effectiveness. The cleaning process of H_2_SO_4_ is represented by reactions (1) and (2) [22]:

Fe_2_O_3_ + 6H^+^ → 2Fe^3+^ + 3H_2_O (1)

Al_2_O_3_ + 6H^+^ → 2Al^3+^ + 3H_2_O (2)

HF and NaOH are notable agents for removing Si impurities [16]. This study used NaOH to eliminate Si impurities in the first two processes, while HF was used instead of NaOH in the third. The reactions for removing SiO_2_ by HF are represented by reactions (3) and (4) [23]:

4HF + SiO_2_ → SiF_4_ + H_2_O (3)

SiF_4_ + 2HF → H_2_SiF_6 (liqiud)_ (4)

After leaching in HF, H_2_SiF_6_ was washed away with distilled water. However, besides SiO_2_, silicate impurities may have existed in the form of Al_2_O_3_.2SiO_2_.2H_2_O [24]; this group of impurities was dissolved in a strong alkaline solution to form Al(OH)_4_^−^, Si(OH)_3_^−^, and H_2_SiO_4_^2−^ ions [25,26]. This could explain the presence of aluminium in the P-III sample, which used the third process P-III (using HF to remove SiO_2_). The reaction mechanism of alkali with silicate compounds is presented in reactions (5) and (6) [27], occurring at temperatures above 400 °C [16], which is below the thermal decomposition of graphite.

Al_2_O_3_.2SiO_2_.2H_2_O + OH^−^ + 5H_2_O → 3Al(OH)_4_^−^ + 2H_4_SiO_4_ (5)

SiO_2_ + 2OH^−^ → H_2_SiO_4_^2−^ (6)

These ions were washed away with distilled water after calcination. However, the immersion in alkali may have generated some sparingly soluble metal hydroxides such as Fe(OH)_3_ and Al(OH)_3_. Therefore, after calcination with alkali, an additional step of leaching in acid was required to convert these hydroxides into more soluble salts. This process is described by reactions (7) and (8) [26]:

Fe(OH)_3_ + 6HCl → FeCl_3_ + 3H_2_O (7)

Al(OH)_3_ + 6HCl → AlCl_3_ + 3H_2_O (8)

In the second process (P-II), H_2_SO_4_ was used instead of HCl; the resulting soluble salts were FeSO_4_ and Al_2_(SO_4_)_3_. All four salts, FeCl_3_, AlCl_3_, FeSO_4_, and Al_2_(SO_4_)_3_, are water-soluble. However, the EDX results of the P-II sample in the second method still showed the presence of Fe and Al elements, whereas the P-I sample from the first method achieved a carbon content of over 99.9%. This suggests that the initial acid pre-treatment step played a crucial role and influenced the outcome of the purifying process.

The research of Jara et al. identified the optimal method for refining graphite to achieve a carbon content of over 99% as the 3-step process H_2_SO_4_/H_2_O_2_–NaOH–HCl; this method is equivalent to the first method in this study [15]. However, the graphite used in this study is in the form of spheres, which is different from the flake graphite, and H_2_O_2_ is a strong oxidizing agent, which may produce SO_2_ gas and cause the spherical particles to swell when combined with H_2_SO_4_. Furthermore, H_2_O_2_ can expand the material into a 2D form [28], which contradicts the expectation of maintaining the spherical shape of graphite particles. Therefore, H_2_O_2_ was not used in this study. From these results, the three-step process of H_2_SO_4_–NaOH–HCl could be considered the most optimal purification method for spherical graphite.

### 3.2. Characterization of Purified Spherical Graphite

X-ray diffraction (XRD), scanning electron microscopy (SEM), and Brunauer–Emmett–Teller (BET) analyses were employed to analyze the structure, morphology, and porosity of the spherical graphite before (SG1) and after (SG2) purification with the optimal process (P-I).

### 3.3. X-ray Diffraction (XRD)

**Figure 6:**
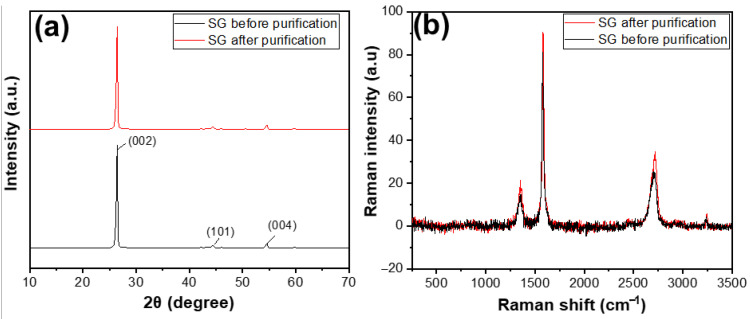
(**a**) XRD patterns and (**b**) Raman spectra of the spherical graphite before and after purification.

Figure 6a shows the XRD results of SG1 and SG2. Before and after purification, both materials exhibited characteristic peaks at an angle of 2θ = 26.5°. Compared with some XRD results of natural graphite studied worldwide, the XRD pattern of the reference material also showed a characteristic peak at 2θ = 26.5° [29]. This indicates that, before treatment, the SG1 was graphite with an hcp structure [30], as evidenced by the characteristic peak at 2θ = 26.5°. After purification, the product SG2 remained graphite and retained its original structure, as there was still a characteristic peak at the corresponding 2θ angle, without any additional peaks. The Raman results, as shown in Figure 6b, clearly indicate that the carbon peaks of SG after purification were higher than that of SG before purification, demonstrating that the carbon content of SG after purification was higher than that of SG before purification.

### 3.4. Scanning Electron Microscopy (SEM)

**Figure 7:**
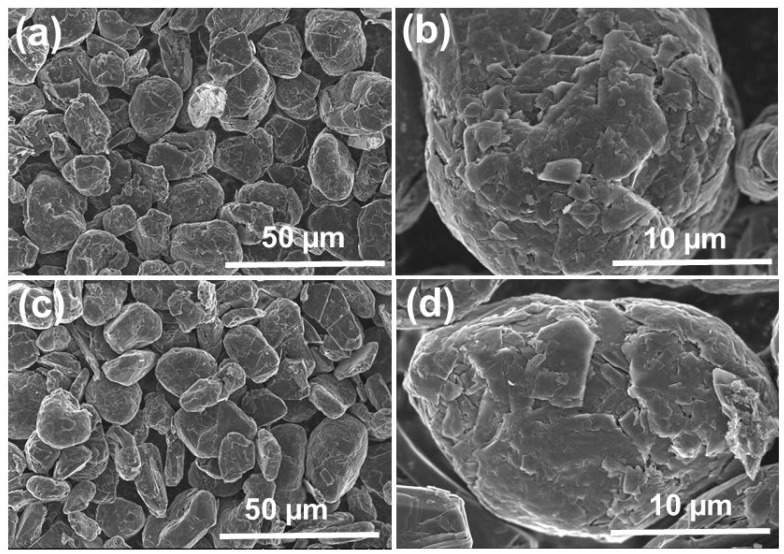
SEM image of the spherical graphite before (**a**,**b**) and after (**c**,**d**) purification.

SEM images of SG1 and SG2 are shown in Figure 7. Before treatment, the graphite (SG1) exhibited spherical shapes with uniform sizes, and the diameter was approximately 20 µm. After cleaning, the graphite particles (SG2) were still spherical and maintained uniform sizes. Additionally, the material’s surface was rougher and contained more holes and defects because of the dissolution of the impurities in the initial spherical graphite. This demonstrates that the purification process cleaned the graphite particles effectively without affecting their spherical shapes.

### 3.5. BET Analysis

**Figure 8:**
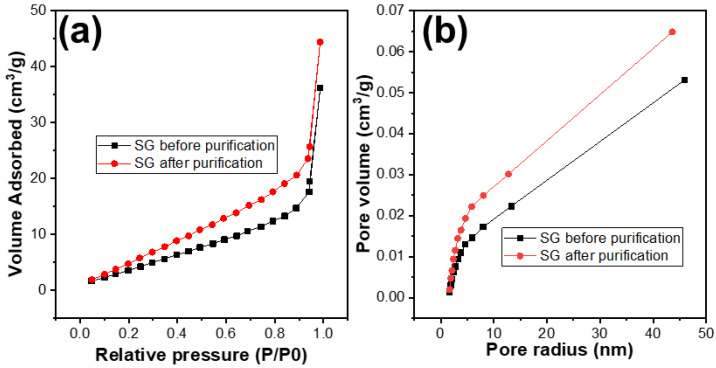
(**a**) N_2_ adsorption and desorption plots and (**b**) the pore size distribution curves of the spherical graphite before and after purification.

The porosity of the SG1 and SG2 were investigated by using the BET analysis. The results are shown in Table 1 and Figure 8. It can be observed that, after treatment, the surface area of the SG2 particles compared to SG1 had improved, with the surface area increasing from 20 m^2^/g to 30 m^2^/g. The distribution of pore sizes did not change significantly and remained at around 1.88 nm. However, the pore volume of SG2 increased from 0.053 cm^3^/g to 0.065 cm^3^/g. This indicates that, after removing impurities, the porosity of the material particles was enhanced, and this improvement in porosity could potentially increase the capacity of the graphite to accommodate Li^+^ ions, or in other words, increase the capacity of the graphite when used as an electrode material for Li-ion batteries.

### 3.6. Electrochemical Investigation

#### 3.6.1. Cyclic Voltammetry (CV)

Cyclic voltammetry was carried out at a scan rate of 0.1 mV/s from 0 V to 3 V in order to investigate the electrochemical behaviour of the graphite before (SG1) and after (SG2) purification. In this experiment, a half-cell model was used. Lithium metal electrodes were used as both reference and counter electrodes; graphite electrodes were working electrodes. The LiPF_6_ 1M solution dissolved in the mixed solvents ethylene carbonate (EC)/diethyl carbonate (DEC)/dimethyl carbonate (DMC) (1:1:1 by volume) was electrolyte.

**Figure 9:**
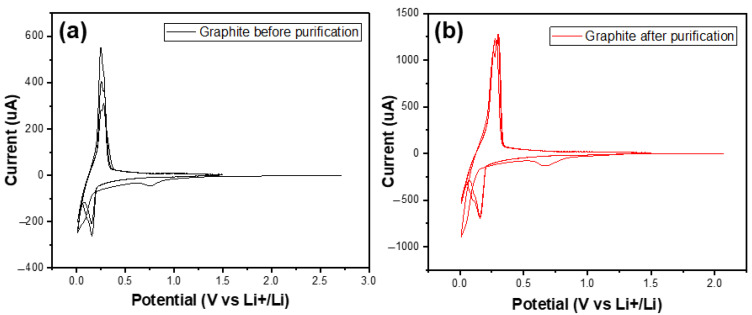
The CV curves of the graphite before (**a**) and after (**b**) purification in the first three cycles.

Figure 9 shows the CV curves of two materials in the first three cycles. In the first cycle, there were small peaks at 0.7 V vs. Li^+^/Li in both graphs of the studied samples. In the following cycles, they no longer appeared. These peaks referred to the decomposition of the electrolytes to build up the SEI (solid electrolyte interface) film [31].

**Figure 10:**
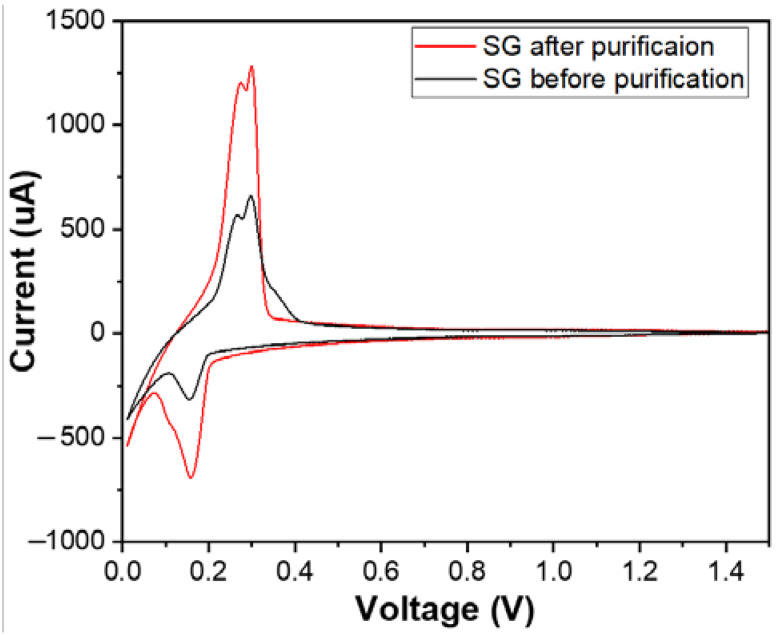
The cyclic voltammetry (CV) curves of the anodes fabricated from the spherical graphite before and after purification.

Figure 10 shows the cyclic voltammetry (CV) curves of materials SG1 and SG2. Both CV curves exhibited complete oxidation peaks (at a potential of 0.26 V) and reduction peaks (at a potential of 0.15 V), and they also had similar shapes to the CV curve or similar electrochemical behaviour of the natural spherical graphite in another study [31]. This demonstrates that the researched materials possess the electrochemical characteristics typical of graphite electrode materials for Li-ion batteries. The intensity of the oxidation and the reduction peak after purification increased, which shows that the purification process enhanced the conductivity of the graphite material.

#### 3.6.2. Galvanic Charge Discharge (GCD)

**Figure 11:**
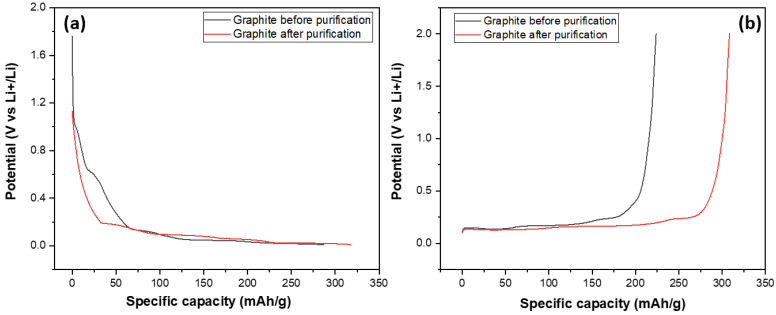
The charge (**a**) and discharge (**b**) curves of the anodes fabricated from the spherical graphite before and after purification.

After investigating their structural and morphological characteristics, SG1 and SG2 were processed into electrodes and assembled into half-cell models for evaluating their electrochemical capacities through galvanic charge–discharge (GCD) measurements at a 0.5 C rate, discharging to 0.001 V and charging to 2 V (this measurement mode is also used in some studies on the electrochemical properties of graphite as an anode material for Li-ion batteries) over 100 cycles. The main target of this experiment was to compare the specific capacities of the graphite before and after purification at the same conditions (charge–discharge current density, mass of electrode); and the results are shown in Figure 11. Figure 11 shows the discharge and charge curves of two graphite samples, SG1 and SG2. The discharge and charge curves of the SG2 material extended to a value of about 350, indicating that the material, after purification, could achieve a specific capacity of 350 mAh/g, which is close to the theoretical capacity of graphite at 372 mAh/g. More importantly, this capacity is much higher than the specific capacity of the untreated material when the SG1 sample reached 280 mAh/g. This can be explained by the BET results mentioned aboved where the increase in the surface area and pore volume enhanced the intercalation/deintercalation of the Li^+^ ions in the graphite, making it easier for the material to absorb or release cation, thereby improving charge/discharge capacity.

**Figure 12:**
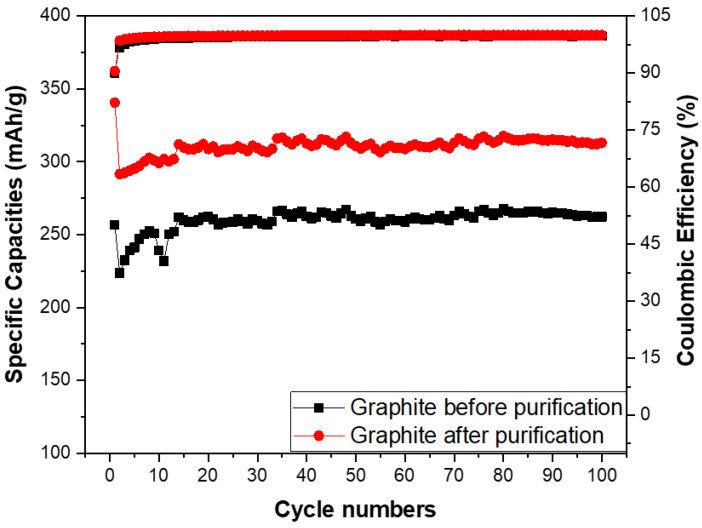
Specific capacities of anodes fabricated from the spherical graphite before and after purification.

The specific capacities and coulombic efficiency of the anodes made from the spherical graphite before and after purification were investigated through over 100 cycles, as shown in Figure 12. The coulombic efficiency of both samples stabilized at approximately 99% from the second cycle onwards, with an initial value of 90% in the first cycle due to the formation of the SEI film. Regarding specific capacity, the SG2 material maintained a stable specific capacity of 300 mAh/g throughout the 100 cycles, while the electrode using SG1 material had a specific capacity below 300 mAh/g. This is lower than the value for the graphite with over 99% carbon content and significantly below the theoretical specific capacity of graphite (372 mAh/g). These results indicate that purifying spherical graphite to achieve a carbon content greater than 99% is essential for its application as an anode material in Li-ion batteries.

## 4. Conclusions

In short, after the research and evaluation of the material characteristics, the results of energy-dispersive X-ray spectroscopy (EDX) measurements indicate that natural spherical graphite, when leached in H_2_SO_4_ and then calcinated with NaOH and neutralised with HCl, could achieve a carbon content from 90% up to 99%. The characteristic evaluation results from XRD, SEM, and BET analysis show that the purified material still maintains its spherical shape and retains the characteristic structure and morphology of graphite. Moreover, the porosity of the purified graphite particles compared to the untreated samples is significantly improved. The electrochemical measurement results demonstrate that cleaned graphite exhibits significantly higher capacity than the spherical graphite without purification processes. The anode made from the purified spherical graphite has a specific capacity of up to 350 mAh/g compared the anode fabricated from the spherical graphite not subjected to the purification process, which had a capacity of 280. These results also prove that purifying graphite to achieve a carbon content of over 99% is essential for its application as an anode material for Li-ion batteries.

## Figures and Tables

**Table 1:** Porous properties of the spherical graphite before and after purification.

Parameters	SG1	SG2
Surface area (m^2^/g)	20.148	30.531
Pore distribution (nm)	1.888	1.889
Pore volume (cm^3^/g)	0.053	0.065

## Data Availability

The original contributions presented in the study are included in the article, further inquiries can be directed to the corresponding author/s.
